# Factors influencing contracting of residents with family doctors in China: a national cross-sectional survey

**DOI:** 10.1186/s12913-024-10606-y

**Published:** 2024-02-15

**Authors:** Ning Zhao, Mei Gu, Jin Li, Haiyan Zhang, Jia Yang

**Affiliations:** 1 School of Public Health, Capital Medical University, Beijing, China; 2 Department of Health Education, Beijing Huairou Hospital of University of Chinese Academy of Sciences, Beijing, China

**Keywords:** Family doctor, Hierarchical medical system, Questionnaire, China

## Abstract

**Background:**

Family doctor contract services (FDCS) have been introduced in China in 2009 [1] and rapidly expanded recently. This study sought to investigate factors that influenced the willingness of Chinese residents to use FDCS.

**Methods:**

We employed multistage stratified and convenience sampling to administer questionnaires to 1455 Beijing, Qinghai, and Fujian residents. The willingness of residents in each province to contract family doctors was analyzed using the chi-square test and binary logistic regression.

**Results:**

The analysis in this study found that the signing rate of family doctors in China was about 27.77%, with differences in the signing up levels in Beijing (13.68%), Fujian (64.49%) and Qinghai (11.22%). In addition, the binary logistic regression results emphasized the relative importance of age, education, medical preference and policy knowledge on the willingness to sign up. Distrust of family doctors’ medical skills (65.7%), not knowing how to contract (47.8%), and not knowing what medical problems can be solved (41.1%) were the top three reasons accounting for the reluctance of residents to contract with family doctors.

**Conclusion:**

Residents from different backgrounds have different willingness to sign up, so the specific circumstances and needs of different groups should be taken into account. In order to increase the signing-up rate, consideration can be given to promoting the family doctor model in Fujian throughout the country. Individual hesitation can be eliminated by increasing the reimbursement rate of health insurance, reducing the out-of-pocket expenses of contracted patients, and providing incentives of certain discounts for consecutive contracted patients.

## Introduction

As an important policy tool to achieve the ambitious goal of “primary health care for all” proposed by the Alma-Ata Declaration, family doctor contract services have been promoted and developed rapidly worldwide over the years [2, 3]. The implementation of family doctor contract services not only provides residents with continuous all-around health services but also plays an important role in promoting the policy goals of primary care, two-way referral, and hierarchical diagnosis. To date, more than 50 countries and regions have implemented family doctor systems, including Canada, Australia, Saudi Arabia and Iran [4–7].

Although different countries have different models of family doctor services, common features exist. England, France, the Netherlands and Indonesia have a “gatekeeper” system in which residents must designate a general practitioner as their family doctor. Residents must visit their family doctor for a diagnosis before they receive a referral [8–11]. In China, this provision is also in force in Hong Kong [12]. At the same time, the number of residents contracted by family doctors is generally set at around 2,000 [13]. There is no mandatory contracting requirement in the United States, Belgium and Japan [14], and residents can voluntarily register with a family doctor and receive health care services [15, 16]. Besides, in Japan, family doctors work in urban or rural hospitals and clinics to provide primary health care by caring for patients in the community in collaboration with a variety of health care professionals [17]. And in Taiwan province, large hospitals in the region cooperate and contract with a number of private or public clinics in the neighbourhood to form a “shared care clinic” [12]. Residents could visit any specialist in community clinics or the outpatient departments of hospitals without a referral [18]. The contracting rate varies from country to country under different family doctor service models. Full coverage of family doctor services has been achieved in the United Kingdom and Cuba [19].

Patients in China have the freedom to choose their hospitals and can even go directly to tertiary hospitals without a referral due to the absence of a strict hierarchical diagnosis and treatment system. This has led to overcrowding of tertiary hospitals and underutilization of primary hospitals. Problems such as difficult and expensive access to medical treatment ensue. In 2013, a pilot FDCS for the elderly (over 65 years of age), pregnant women, children (0–6 years of age) and patients with chronic diseases was introduced in some regions of China. Residents can contract with a preferred family medicine team, which usually consists of general practitioners, nurses and public health doctors. The contract is usually for one year and residents can change the contracted doctor if they are not satisfied with the service the following year [20]. Contracted residents are encouraged to visit their family doctors for health problems, but patients retain the freedom to choose their preferred health institutions [21]. One of the key elements of FDCS is providing health checkups, consultations, and chronic disease management for residents with chronic diseases. Although the service models vary from region to region, they are all based on the “gatekeeper” system in primary care [1, 21–23]. After signing up, residents only need to pay a few dollars ($4.2 per person per year in Beijing, $2.8 per person per year in Fujian and Qinghai) to receive free basic medical services. Appointments for specialists in higher-level hospitals can be made several weeks in advance through family doctors, for example, 10 days in advance in Fujian. In addition, residents can receive an increase in the reimbursement rate for health insurance, with Fujian increasing the reimbursement rate by 5% points from the original rate.

In 2016, seven ministries and commissions under the State Council jointly issued the “Guiding Opinions on Promoting Family Doctor Contract Services”, marking the full launch of FDCS in China. The “Guiding Opinions on Regulating the Management of Family Doctor Contract Services” released in 2018, improved the quality and efficiency of FDCS. In 2022, the Chinese Health Council issued the “Guidance on Promoting the High-Quality Development of Family Doctor Contract Services”, requiring that by 2035, the coverage rate of contracted services should reach more than 75%, with basic full coverage of households, which further promoted the high-quality development of FDCS. China has been issuing new policies related to FDCS in recent years, indicating the importance it attaches to FDCS, with the ultimate goal of increasing the signing rate and meeting the multi-level and diversified health service needs of residents. Accordingly, exploring the factors that influence the contract services of family doctors is an important part of improving the signing rate.

In recent years, many studies have focused on exploring the factors influencing residents’ willingness to sign up, focusing on individual characteristics, distance, quality of service, satisfaction, perception, and medical experience [24–29]. Some studies have only focused on a particular province or city, limiting the generalizability of their findings, while the actual signing rates of cross-provincial residents and residents’ willingness to sign up have been largely understudied [30, 31]. Current evidence suggests different factors influence willingness to sign up in regions, including health care resources, service levels, health literacy, etc [24, 31]. As the family doctor system starts to be promoted nationwide, little is currently known about how residents in different regions respond to FDCS. Current research focuses mostly on the influence of personal factors such as age, education level, personal income (per month) and history of chronic diseases, as well as the influence of factors such as publicity, quality of primary health care services and the ability of family doctors to provide the services on family doctor contracting services, but there are fewer studies on the influencing factors of contracting services for residents in different regions [1, 3, 32, 33].

In this study, three provinces were selected based on regional distribution and economic development level to investigate and understand the actual situation of residents contracting family doctors nationwide. We tried to answer the following questions: (1) Are residents aware of FDCS? (2) What is the actual signing rate of residents? (3) What factors may influence residents’ contracting with family doctors? The solution to these questions will improve residents’ cognition of FDCS, optimize the content of FDCS, foster family doctors to provide better services and improve the signing rate of family doctors. Our findings will provide suggestions for further optimization of policies related to FDCS and lay the groundwork for promoting the development of high-quality FDCS.

## Methods

### Study design and data sources

Multistage sampling was conducted to select the participants following the four steps below.

First, three provinces were selected based on the progress of the implementation of the family doctor system, regional distribution and level of economic development in each region (North: Beijing; Southeast: Fujian; Central and West: Qinghai). Among them, Beijing is one of the earliest batch of cities in the country to pilot the FDCS (2010). Since 2014, Fujian has formed a unique “co-management” family doctor contract model that combines specialists from large hospitals, primary general practitioners and health managers, with chronic diseases as the breakthrough. As an underdeveloped inland region, Qinghai began to fully implement FDCS in 2017, making it an important measure for health poverty alleviation.

Next, two cities were selected in each province based on the implementation progress of the family doctor system, for a total of six cities. The formula used to calculate the sample size of residents in each city is $\text{n}=\frac{{\text{Z}}^{2}\text{P}(1-\text{P})}{{\text{E}}^{2}}$ [1, 22, 34], which yielded a sample size of *n* = 263 at Z = 1.96, *P* = 22% (according to the report data of the Administrative Departments of Public Health), and allowable error E = 0.05. Accordingly, we selected 300 residents in each city [21].

Third, two tertiary hospitals, two secondary hospitals, and two community health centers were selected in each city with the support of the China Hospital Association. The sample size of tertiary hospitals, secondary hospitals, and community health centers in each city was 100.

Subsequently, residents were surveyed using a convenience sampling method, and 50 were selected from each hospital. A total of 1807 questionnaires were distributed, and after excluding residents who were unsure whether they were contracted or not and invalid questionnaires, a total of 1455 residents were included in the study sample, with an effective rate of 80.52%.

**Fig. 1:**
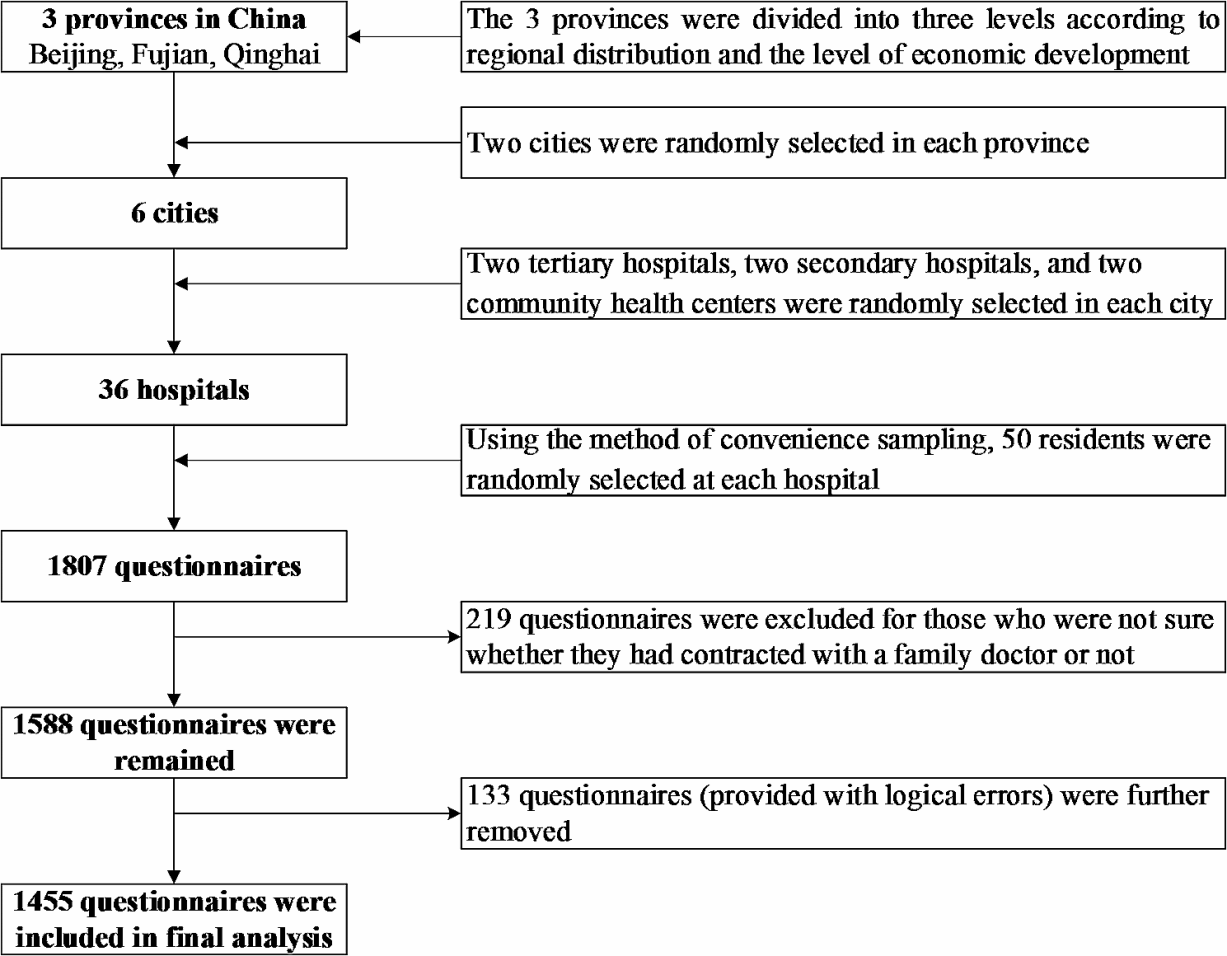
Flowchart of the sampling method

The sample size selection process was showed in Fig. 1.

The questionnaire was created based on a literature review and professional advice [22, 35–39]. Following the completion of the questionnaire design, we looked for respondents to carry out a pre-survey to evaluate the validity and reliability of the questionnaire. We then amended the questionnaire based on the findings. The questionnaire’s structure and content were decided upon after numerous iterations. Finally, an electronic version of the survey questionnaire was obtained. To guarantee the response rate and authenticity of the data, respondents were offered survey incentives during face-to-face interviews with trained interviewers. Only residents older than 18 were included to ensure the data’s accuracy. Before handing out the questionnaire, we stated the study purpose and content to all respondents, obtained their informed consent, and ensured that their privacy would be protected. This study was conducted from March to May 2019. According to the study design, only parts of the questions in the questionnaire were included as variables.

### Dependent variable

In this study, the dependent variable was the willingness of residents to contract a family doctor, assessed by the question, “Have you contracted with a family doctor?” with only two possible answers: (a) Yes; (b) No.

### Independent variables

The independent variables consisted of three main components. The first part comprised sociodemographic characteristics, including province, gender, age, education, annual household income, average monthly medical expense, and chronic disease status [22, 35]. The second part assessed the degree of understanding of the policy, including the understanding of the community first visit policy, which means that primary health care facilities serve as “gatekeeper” and secondary and tertiary hospitals provide specialised care [35], and the family doctor contract services policy [36, 37]. The third part evaluated the attitude toward the policy, including the choice of medical treatment when suffering from common diseases [38], the attitude toward the hierarchical medical system and the recognition of the hierarchical medical system [39].

### Statistical analysis

Statistical analysis was performed using SPSS 21.0 software for data analysis. Descriptive statistics (frequencies and percentages) were used to describe the sociodemographic characteristics of the residents. The chi-square test was used to compare the basic characteristics and willingness to contract of residents in Beijing, Fujian and Qinghai, and binary logistic regression was used to analyze the influencing factors of residents’ willingness to contract in the three provinces respectively. A *P*-value < 0.05 was statistically significant.

## Results

### Comparison of basic characteristics of residents in Beijing, Fujian and Qinghai

As shown in Table 1, the difference in the distribution of Beijing, Fujian and Qinghai residents in terms of gender and household registration status is not statistically significant (*P* > 0.05). Compared with residents of Beijing and Qinghai, residents of Fujian are older (4.91% vs. 4.62% vs. 4.13% for those over 65 years old), more educated (31.54% vs. 28.13% vs. 27.17% for those with bachelor’s degree or above), and have lower average monthly medical expenses (15.89% vs. 24.86% vs. 20.08% for those over 800 yuan), Fewer patients chose secondary and higher level medical institutions for chronic diseases (34.11% vs. 40.46% vs. 34.65%), and knew more about the family doctor contract policy (90.19% vs. 19.85% vs. 29.53%). Compared with residents of Beijing and Fujian, residents of Qinghai have a larger floating population (9.06% vs. 7.71% vs. 8.18% for 1 year or less), more patients with chronic diseases (100.00% vs. 91.71% vs. 99.53%), and fewer patients with common diseases choosing second-level or higher medical institutions (19.49% vs. 21.39% vs. 20.09%), less aware of the policy of the community first visit policy (6.30% vs. 14.64% vs. 89.49%), less recognizing the community first visit policy (42.91% vs. 85.16% vs. 50.23%), more supportive of the hierarchical medical system (45.87% vs. 44.70% vs. 39.49%), and more recognizing the hierarchical medical system(72.64% vs. 55.88% vs. 69.39%).There were differences in the willingness to sign up among residents of the three provinces, and the willingness to sign up among residents of Fujian (64.49%) was higher than that of residents of Beijing (13.68%) and Qinghai (11.22%), and the difference was statistically significant (χ^2^ = 408.413, *P* < 0.001).

**Table 1:** Contracting status of FDCS among residents

Variables	Province			Cases	χ^2^	*P*
	Beijing	Fujian	Qinghai			
Total	519	428	508	1455		
**Gender**					1.034	0.596
Male	229	202	226	657		
Female	290	226	282	798		
**Age (years)**					15.551	**0.016**
< 45	323	219	289	831		
45–54	117	120	117	354		
55–64	55	68	81	204		
≥ 65	24	21	21	66		
**Education**					11.588	**0.021**
Junior or below	21	22	43	86		
Senior high school	352	271	327	950		
Bachelor or above	146	135	138	419		
**Household status**					5.294	0.258
Downtown	157	114	131	402		
Suburbs	118	94	103	315		
Outside the city	244	220	274	738		
**Length of residence**					9.561	**0.049**
Less than 1 year	40	35	46	121		
1–2 years	81	92	112	285		
More than 2 years	398	301	350	1049		
**Average monthly medical expense**					12.601	**0.013**
≤CNY 300 (US$ 42)	91	75	95	261		
CNY 301–800 (US$ 42–112)	299	285	311	895		
>CNY 800 (US$ 112)	129	68	102	299		
**Chronic disease status**					72.743	**0.000**
Yes	476	426	508	1410		
No	43	2	0	45		
**Medical institution of choice for common diseases**					31.655	**0.000**
Pharmacy	117	142	190	449		
Community health service center	253	181	196	630		
Secondary Hospital	84	69	76	229		
Tertiary Hospitals	27	17	23	67		
Others	38	19	23	80		
**Medical institution of choice for chronic diseases**					34.274	**0.000**
Pharmacy	102	131	176	409		
Community health service center	193	139	142	474		
Secondary Hospital	129	99	119	347		
Tertiary Hospitals	81	47	57	185		
Others	14	12	14	40		
**Understanding of the family doctor contract services policy**					55.945	**0.000**
Yes	103	386	150	639		
No	416	42	358	816		
**Signing of family doctors**					408.413	**0.000**
Yes	71	276	57	404		
No	448	152	451	1051		
**Understanding of the community first visit policy**					18.996	**0.000**
Yes	76	383	32	491		
No	443	45	476	964		
**Attitudes toward the community first visit policy**					355.900	**0.000**
Not recognize	77	213	290	580		
Moderate	77	109	124	310		
Partly recognize	238	67	69	374		
Completely recognize	127	39	25	191		
**Attitudes toward the hierarchical medical system**					37.534	**0.000**
Support	232	169	233	634		
Worry	43	61	68	172		
Still many problems	105	69	122	296		
Not clear	139	129	85	353		
**Recognition of the hierarchical medical system**					57.062	**0.000**
Not recognize	45	35	31	111		
Moderate	184	96	108	388		
Partly recognize	241	207	253	701		
Completely recognize	49	90	116	255		

### Factors associated with residents contracting with family doctors

Logistic regression analyses were conducted for each of the three provinces, the logistic regression models used contracting status (signed and non-signed) as the dependent variable. The results are shown in Table 2.

**Table 2:** Binary logistic regression analysis of the factors associated with contracting with family doctors by province

	Beijing (*N* = 519) (R^2^ = 0.346 *P* < 0.001)					Fujian (*N* = 428) (R^2^= 0.200, *P* < 0.001)					Qinghai (*N* = 508) (R^2^ = 0.169, *P* < 0.05)				
	N	β	S.E	OR	*P*	N	β	S.E	OR	*P*	N	β	S.E	OR	*P*
Sex															
Male	229	Reference				202	Reference				226	Reference			
Female	290	-0.075	0.360	0.928(0.458–1.88)	0.836	226	-0.561	0.232	0.571(0.362-0.9)	**0.016**	282	0.234	0.320	1.264(0.675–2.365)	0.464
**Age**															
< 45	323	Reference				219	Reference				289	Reference			
45–54	117	0.130	0.421	1.138(0.499–2.597)	0.758	120	0.604	0.267	0.547(0.324–0.922)	**0.024**	117	-0.501	0.411	0.606(0.271–1.354)	0.222
55–64	55	1.295	0.519	3.651(1.319–10.105)	**0.013**	68	0.665	0.330	0.514(0.269–0.983)	**0.044**	81	0.195	0.397	1.216(0.558–2.648)	0.623
≥ 65	24	1.572	0.804	4.817(0.995–23.314)	0.051	21	0.883	0.702	2.418(0.611–9.562)	0.208	21	-0.600	0.854	0.549(0.103–2.928)	0.483
**Education**															
Junior or below	21	Reference				22	Reference				43	Reference			
Senior high school	352	0.300	0.852	1.350(0.254–7.163)	0.725	271	0.985	0.532	2.679(0.945–7.594)	0.064	327	0.464	0.665	1.590(0.432–5.857)	0.486
Bachelor or above	146	0.841	0.867	2.319(0.424–12.682)	0.332	135	1.316	0.558	3.728(1.249–11.126)	**0.018**	138	0.795	0.696	2.215(0.566–8.668)	0.253
**Household status**															
Downtown	157	Reference				114	Reference				131	Reference			
Suburbs	118	-0.211	0.261	0.810(0.360–1.822)	0.610	94	-0.967	0.344	2.630(1.339–5.166)	**0.005**	103	-0.017	0.485	0.983(0.3804–2.544)	0.973
Outside the city	244	-1.304	10.54	0.271(0.123–0.596)	**0.001**	220	-0.607	0.278	1.835(1.064–3.163)	**0.029**	274	0.610	0.394	1.840(0.850–3.981)	0.122
**Length of residence**															
Less than 1 year	40	Reference				35	Reference				46	Reference			
1–2 years	81	2.039	1.284	7.685(0.62-95.217)	0.112	92	0.322	0.449	1.380(0.573–3.323)	0.473	112	0.622	0.724	1.863(0.451-7.700)	0.390
More than 2 years	398	2.021	1.249	7.549(0.653–87.298)	0.106	301	0.433	0.400	1.542(0.704–3.377)	0.278	350	0.879	0.655	2.409(0.667–8.703)	0.180
**Chronic disease status**															
Yes	476	Reference				426	Reference				508	Reference			
No	43	-1.463	0.630	0.232(0.067–0.796)	**0.020**	2	-21.368	28420.757	0.000	0.999	0	-			
**Average monthly medical expense**															
≤CNY 300 (US$ 42)	91	Reference				75	Reference				95	Reference			
CNY 301–800 (US$ 42–112)	299	0.506	0.496	1.659(0.628–4.383)	0.307	285	0.855	0.310	2.351(1.280–4.317)	**0.006**	311	0.014	0.390	1.014(0.472–2.178)	0.971
>CNY 800 (US$ 112)	129	-0.347	0.621	0.707(0.209–2.385)	0.576	68	1.101	0.400	3.008(1.373–6.590)	**0.006**	102	-0.690	0.549	0.502(0.171–1.472)	0.209
**Medical institution of choice for common diseases**															
Pharmacy	117	Reference				142	Reference				190	Reference			
Community health service center	253	0.327	0.428	1.386(0.599–3.209)	0.446	181	0.491	0.270	1.633(0.962–2.774)	0.069	196	0.662	0.362	1.939(0.953–3.943)	0.068
Secondary Hospital	84	-0.390	0.690	0.677(0.175–2.617)	0.572	69	-0.236	0.346	0.790(0.401–1.557)	0.496	76	-0.443	0.599	0.642(0.198–2.078)	0.460
Tertiary Hospitals	27	0.471	0.745	1.601(0.372–6.895)	0.528	17	-0.418	0.565	0.658(0.217–1.993)	0.460	23	1.191	0.616	3.290(0.983–11.014)	0.053
Others	38	0.897	0.675	2.453(0.654–9.201)	0.183	19	0.385	0.599	1.470(0.455–4.751)	0.520	23	-0.783	1.101	0.457(0.053–3.955)	0.477
**Medical institution of choice for chronic diseases**															
Pharmacy	102	Reference				131	Reference				176	Reference			
Community health service center	193	1.191	0.526	3.291(1.173–9.236)	**0.024**	139	-0.056	0.301	0.946(0.524–1.707)	0.853	142	0.703	0.396	2.021(0.929–4.394)	0.076
Secondary Hospital	129	0.254	0.596	1.289(0.401–4.149)	0.670	99	0.068	0.320	1.071(0.572–2.005)	0.831	119	0.690	0.424	1.994(0.869–4.580)	0.104
Tertiary Hospitals	81	-0.126	0.685	0.882(0.230–3.379)	0.855	47	-0.725	0.409	0.485(0.217–1.080)	0.076	57	-0.620	0.687	0.538(0.140–2.066)	0.366
Others	14	2.915	0.957	18.456(2.828-120.429)	**0.002**	12	0.662	0.775	1.94(0.425–8.862)	0.393	14	1.814	0.724	6.133(1.484–25.342)	**0.012**
**Understanding of the family doctor contract services policy**															
Yes	103	Reference				386	Reference				150	Reference			
No	416	0.927	0.398	2.526(1.157–5.513)	**0.020**	42	-1.052	0.397	0.349(0.160–0.760)	**0.008**	358	-0.277	0.348	0.758(0.383-1.500)	0.426
**Understanding of the community first visit policy**															
Yes	76	Reference				383	Reference				32	Reference			
No	443	-1.058	0.445	0.347(0.145–0.830)	**0.017**	45	0.125	0.376	1.133(0.543–2.366)	0.739	476	0.163	0.667	1.177(0.318–4.355)	0.807
**Attitudes toward the community first visit policy**															
Not recognize	77	Reference				213	Reference				290	Reference			
Moderate	77	2.905	0.884	18.263(3.231-103.219)	**0.001**	109	0.210	0.289	1.234(0.700-2.176)	0.468	124	0.705	0.368	2.025(0.985–4.161)	0.055
Partly recognize	238	2.190	0.823	8.938(1.783–44.815)	**0.008**	67	-0.126	0.329	0.882(0.463–1.68)	0.703	69	0.090	0.482	1.094(0.425–2.815)	0.853
Completely recognize	127	2.111	0.848	8.257(1.567–43.52)	**0.013**	39	-0.146	0.405	0.864(0.391–1.911)	0.718	25	-1.004	1.073	0.366(0.045–3.004)	0.350
**Attitudes toward the hierarchical medical system**															
Support	232	Reference				169	Reference				233	Reference			
Worry	43	-0.009	0.873	0.991(0.179–5.488)	0.992	61	0.228	0.367	1.257(0.612–2.582)	0.534	68	-0.059	0.518	0.943(0.342–2.601)	0.910
Still many problems	105	0.024	0.449	1.025(0.425–2.472)	0.957	69	-0.333	0.329	0.717(0.376–1.366)	0.311	122	0.706	0.377	2.025(0.967–4.243)	0.062
Not clear	139	0.596	0.424	1.815(0.790–4.17)	0.160	129	-0.030	0.287	0.971(0.553–1.704)	0.917	85	0.710	0.423	2.035(0.888–4.662)	0.093
**Recognition of the hierarchical medical system**															
Not recognize	45	Reference				35	Reference				31	Reference			
Moderate	184	0.647	0.721	1.910(0.464–7.855)	0.370	96	-0.107	0.472	0.898(0.356–2.265)	0.820	108	-0.141	0.784	0.868(0.187–4.041)	0.857
Partly recognize	241	1.141	0.721	3.129(0.762–12.857)	0.114	207	-0.306	0.439	0.736(0.312–1.741)	0.486	253	0.255	0.713	1.290(0.319–5.213)	0.721
Completely recognize	49	1.394	0.903	4.030(0.686–23.661)	0.123	90	0.472	0.495	1.603(0.608–4.227)	0.340	116	0.216	0.740	1.241(0.291–5.294)	0.770

R^2^ = Nagelkerkes coefficient of determination

The willingness of both Beijing and Fujian residents to sign up may be related to age, household registration and knowledge of the family doctor contract policy. Among them, Beijing residents’ willingness to sign up may be related to chronic disease status, knowledge of the community first visit policy, and attitude towards the community first visit policy. Fujian residents’ willingness to sign up may be related to gender, education, and average monthly medical expenditure. In addition, Beijing and Qinghai residents’ willingness to sign up may also be related to the choice of chronic diseases.

In the modeling of the signing situation of Beijing residents, the willingness to sign up was higher among those aged 55 to 64 than other age groups (OR = 3.651, *P* < 0.05). Compared with local downtown residents, residents from out of the city were less willing to sign up for a family doctor (OR = 0.271, *P* < 0.01). Residents who did not suffer from chronic diseases were less willing to sign up (OR = 0.232, *P* < 0.05). Those residents who went to community health service center or other places to obtain treatment for their chronic diseases were more willing to sign up for a family doctor than those in the reference group (OR = 3.291, *P* < 0.05; OR = 18.456, *P* < 0.01). In addition, residents who were unaware of the family doctor contract services policy were less likely to sign up compared to those who were aware of it (OR = 2.526, *P* < 0.05). In contrast, residents who recognized the policy of community first care to varying degrees were more likely to sign up for a family doctor (OR = 18.263, *P* < 0.01; OR = 8.938, *P* < 0.01; OR = 8.257, *P* < 0.05).

In the model of Fujian residents’ signing situation, the willingness of female residents to sign up was lower than that of male residents (OR = 0.571, *P* < 0.05).The willingness to sign up of people aged 45 to 54 and 55 to 54 was higher than that of other age groups (OR = 0.547, *P* < 0.05; OR = 0.514, *P* < 0.05). Residents with bachelor’s degree or above had higher willingness to sign up than those with other education (OR = 3.728, *P* < 0.05). Residents in the suburbs and outside the city were more reluctant to sign up for a family doctor than residents in the downtown (OR = 2.630, *P* < 0.01; OR = 1.835, *P* < 0.05). The results also found that residents with average monthly medical expenditures of ¥301 to ¥800 and more than ¥800 had a higher willingness to sign up than those with less than ¥300 (OR = 2.351, *P* < 0.01; OR = 3.008, *P* < 0.01). Compared with residents who were aware of the family doctor contract services policy, those who were not aware had lower willingness to sign up (OR = 0.349, *P* < 0.01).

In the model of signing situation of Qinghai residents, residents who chose other institutions were more willing to sign up for family doctors compared to those who went to pharmacies to obtain chronic disease treatment (OR = 6.133, *P* < 0.05).

**Fig. 2:**
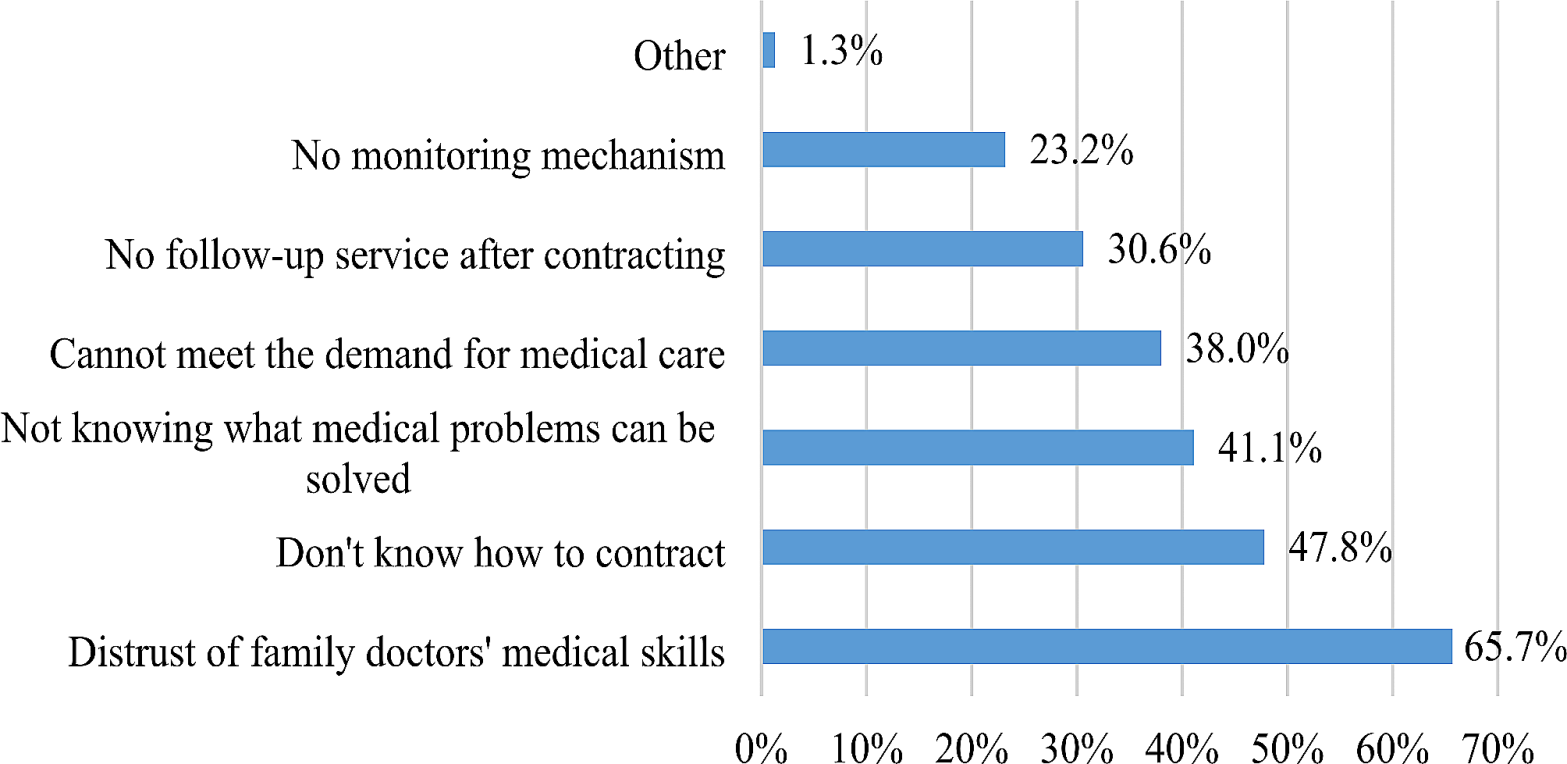
Reasons for residents who did not contract with family doctors

Figure 2 shows the reasons why residents were reluctant to contract with family doctors. Distrust of family doctors’ medical skills (65.7%), not knowing how to contract (47.8%), and not knowing what medical problems can be solved (41.1%) were the top three reasons accounting for the reluctance of residents to contract with family doctors.

## Discussion

In this study, 27.77% of the residents contracted with a family doctor, consistent with previous studies focusing on the signing rate in regions of China [40, 41]. This result largely met the expected target set by the Chinese government in 2017 (30%). Compared to Beijing (13.68%) and Qinghai (11.22%), residents of Fujian (64.49%) exhibited a higher signing rate, which may be attributed to the fact that Fujian provides “co-management” contract services with the cooperation of specialists, general practitioners, and health managers, encouraging residents suffering from common diseases and multi-morbidities to go to community hospitals, unlike the contract services model in other regions [42, 43]. On the other hand, Fujian’s chronic disease-focused contract services are better than those in other regions. For example, family members of residents with diabetes or hypertension are also eligible for screening and intervention services, and the reimbursement rate for residents’ visits to community hospitals is increased by 5%. Compared with other provinces, this model of FDCS is widely used by residents.

Qinghai has a lower sign-up rate. One possible reason for this is that Qinghai was the latest to implement among the three provinces. Another reason may be due to Qinghai’s location in western China, the low quality of primary care services [44], and the lack of resident support for a hierarchical medical system. FDCS in China are usually carried out in primary healthcare institutions, but according to the latest data, in 2021, there were 11,644 registered general practitioners in Fujian, while there were only 1,686 in Qinghai, with 27,463 primary healthcare institutions in Fujian compared to 6,015 in Qinghai [45, 46]. Therefore, the willingness of residents to sign up may be influenced by the healthcare resources in different provinces.

Absolutely, knowledge of the policy or lack thereof is also an important factor that affects residents. Compared with Fujian residents (90.19%), Beijing residents (19.85%) and Qinghai residents (29.53%) were less aware of the family doctor contract services policy, which was similar to a previous study in Shenzhen, China [47], suggesting that the understanding of FDCS may be lower than their utilization rate [48], which may be attributed to poor promotion of FDCS. During the survey conducted to investigate why residents are not willing to sign up with family doctors, “don’t know how to sign a contract” and “not knowing what medical problems can be solved” ranked second and third, respectively, suggesting that residents’ awareness of FDCS should be further improved through a public sensitization campaign.

Consistent with published studies [1, 41], we found that age was positively associated with willingness to contract. An alternative explanation would be that older residents have a higher prevalence of chronic diseases [49] Another explanation was that older residents had a higher utilization of primary care services, especially older residents with multiple chronic diseases were more likely to use long-term care services compared to older residents without multiple chronic diseases [50, 51]. Thus, older residents are most likely to benefit from FDCS.

This study also showed that residents with a higher educational background were more likely to contract, which was consistent with the published study [41]. It is easy to understand: more educated residents have a higher level of health knowledge and are more concerned with information about health benefits [40, 52, 53]. Therefore, residents with high education levels have a higher acceptance of health knowledge, can better understand the policies related to contracted services, and have a higher willingness to use contracted services.

Moreover, results also revealed that medical preference was a relevant influencing factor. Residents who chose primary care for their medical treatment had a higher willingness to sign up for a family doctor [54]. To some extent, this finding indicated that this group of residents has a greater desire for primary care services and a higher demand for health monitoring and management, which is consistent with the duties of family doctors [34]. To promote the implementation of FDCS, the government should prioritize residents with a high demand for community health services as key populations [55].

### Strengths and limitations

This study is a multi-provincial, large-sample research study that improves generalizability in a Chinese setting. Meanwhile, this study has some limitations. First, the cross-sectional design of this study limits the ability to infer causality between influencing factors and residents’ willingness. Besides, this study did not include other factors that affect residents’ willingness, such as residents’ psychological status. In addition, our study was only based on the demand-side perspective.

## Conclusions

The analysis in this study found that the signing rate of family doctors in China is about 27.77%, with variability in the level of signing up among the three provinces of Beijing, Fujian and Qinghai. In addition, these findings emphasize the relative importance of age, education, medical preference, and knowledge of policies on willingness to sign up. To sum up, our study has some practical value and theoretical implications.

These findings may be helpful to Chinese health policy makers in some ways. As a matter of fact, the policies and effects of family doctors in Beijing, Fujian and Qinghai are not the same. Residents from different backgrounds have different willingness to sign up, so the specific circumstances and needs of different groups should be taken into account. First, residents’ awareness of FDCS warrants further improvement. Mass communication tools should be harnessed to strengthen family doctor services promotion. Besides, more efforts should be undertaken to improve residents’ health literacy and change their health concepts to promote their initiative to sign up with family doctors. Furthermore, it is essential to promote the implementation of the hierarchical medical system, improve the level of services provided by primary medical institutions, and gain the trust of the residents. Last but not least, the differences in the level of contract services between provinces should be addressed to achieve geographical equity in FDCS. In order to increase the signing-up rate, consideration can be given to promoting the family doctor model in Fujian throughout the country. However, even in Fujian, there are some individuals who insist on not signing up. Further incentives could be considered to remove individuals’ hesitation. Therefore, the leverage of health insurance should be utilized to increase the reimbursement rate of health insurance for contracted residents and reduce out-of-pocket expenses. For patients who sign up consecutively, incentives can be given to reduce the sign-up fee.

## Data Availability

The original contributions presented in the study are included in the article material, the data for this study are part of the overall project and so are not publicly available. Access to the datasets of this study can be directed to the corresponding authors.

## Abbreviations

FDCS: Family doctor contract services
